# Analysis of Motor and Perceptual–Cognitive Performance in Young Soccer Players: Insights into Training Experience and Biological Maturation

**DOI:** 10.3390/sports14010022

**Published:** 2026-01-05

**Authors:** Afroditi Lola, Eleni Bassa, Sousana Symeonidou, Georgia Stavropoulou, Anastasia Papavasileiou, Kiriakos Fregidis, Marios Bismpos

**Affiliations:** 1 School of Physical Education and Sport Science, Aristotle University of Thessaloniki, 57001 Thessaloniki, Greece; 2 School of Physical Education and Sport Science at Serres, Aristotle University of Thessaloniki, 62500 Serres, Greece; 3 School of Philosophy and Education, Aristotle University of Thessaloniki, 54124 Thessaloniki, Greece; 4 School of Health Sciences, Faculty of Life and Health Sciences, Frederick University, Nicosia 1036, Cyprus

**Keywords:** youth soccer, motor development, biological maturity, training age, perceptual-cognitive skills, athlete health, well-being, physical activity

## Abstract

**Background/Objectives:** This cross-sectional study examined how training age, chronological age, and biological maturity influence motor and perceptual–cognitive performance in youth soccer players, with relevance for health and well-being through sport participation. **Methods:** Forty-one male athletes (age = 14.86 ± 0.81 years) completed a two-day field-based assessment following a holistic framework integrating motor (sprinting, jumping, and agility) and perceptual–cognitive components (psychomotor speed, visuospatial working memory, and spatial visualization). Biological maturity was estimated using the maturity offset method. **Results:** Regression analyses showed that biological maturity and training age significantly predicted motor performance, particularly sprinting, jumping, and pre-planned agility, whereas chronological age was not a predictor. In contrast, neither maturity nor training experience influenced perceptual–cognitive skills. Among cognitive measures, only psychomotor speed significantly predicted reactive agility, emphasizing the role of rapid information processing in dynamic, game-specific contexts. **Conclusions:** Youth soccer training should address both physical and cognitive development through complementary strategies. Physical preparation should be tailored to maturity status to ensure safe and progressive loading, while systematic training of psychomotor speed and decision-making should enhance reactive agility and game intelligence. Integrating maturity and perceptual–cognitive assessments may support individualized development, improved performance, and long-term well-being.

## 1. Introduction

Optimal athletic development in youth depends on the interplay of physical growth, biological maturation, perceptual development, and sport-specific training experience. During adolescence, athletes undergo rapid and asynchronous changes that impact performance, with implications not only for sport but also for overall health and well-being [1,2]. Understanding these factors is essential for designing age-appropriate training, long-term athlete development models, and talent identification systems that also promote lifelong engagement in physical activity [3].

Maturation is a key determinant of adolescent athletic performance. Peak Height Velocity (PHV), the period of maximum growth in stature, is commonly used as a marker of biological maturity and has been linked to gains in strength, speed, and power [4,5]. Athletes near or beyond PHV typically show enhanced coordination, muscle mass, and anaerobic capacity, offering a temporary performance advantage over less mature peers [6]. However, these advantages do not necessarily predict long-term potential, underscoring the need to consider maturity in training and competition [7]. Moreover, not all motor skills are equally influenced by maturation; for example, change of direction asymmetries appear unaffected [8].

Perceptual–cognitive development is equally crucial. Executive functions such as working memory, inhibitory control, and cognitive flexibility mature through adolescence and underpin effective decision-making in soccer [9,10]. Players must interpret complex cues, anticipate opponents, and respond rapidly, with these skills proving fundamental to tactical adaptation and situational awareness [11,12]. Research shows that elite youth soccer players outperform sub-elite peers in anticipation, decision-making, and pattern recognition, suggesting that perceptual–cognitive expertise distinguishes higher-level players [13,14]. Recent technological approaches, such as virtual reality-based perceptual–cognitive training and video-based anticipation tasks, have shown promising results in enhancing decision-making and visual search in youth sport [15,16]. Such expertise develops through deliberate practice and accumulated sport-specific experience [17,18]. Training age, namely the years of structured practice, has been consistently linked to superior perceptual-motor performance [10,19,20]. In addition, ecological and constraints-led approaches highlight how structured perceptual–cognitive interventions can support the development of decision-making and anticipation in young athletes [21].

Despite their importance, perceptual–cognitive skills are often overlooked in talent identification, which tends to prioritize physical and technical traits [22]. This bias may favor early maturers while neglecting late developers with strong perceptual–cognitive potential [23]. Emerging methods, including sport-specific cognitive tests and virtual reality, are helping provide ecologically valid assessments of these abilities [19,24].

A more integrative approach is therefore needed, one that considers growth, maturation, perceptual development, and training history to support both immediate performance and long-term success. Beyond sport, such approaches can foster physical literacy, cognitive stimulation, and psychosocial well-being, thereby contributing to healthier and more active lifestyles [25]. The present study addresses this by examining how training age, chronological age, and biological maturity relate to cognitive and motor skills in young male soccer players. We hypothesize the following: (a) these factors are significantly associated with perceptual skills, (b) they are associated with motor skills, and (c) they can serve as predictors of perceptual and motor performance.

## 2. Materials and Methods

### 2.1. Participants

A total of 41 male youth athletes (N = 41), all actively engaged in competitive soccer, voluntarily participated in the study. The participants were members of local soccer clubs and trained regularly under structured coaching programs. Their ages ranged from 12.52 to 16.71 years (14.86 ± 0.81), while their soccer training age ranged from 1 to 12 years of systematic training experience (7.43 ± 3.09). Age categories were divided into U14 and U16. For training experience, participants were divided into two groups (1–6 years and 7–12 years of training), a split guided by the distribution of the data and quartiles to ensure relatively balanced group sizes and a clear distinction in training experience. Beyond the statistical rationale, the cut-off is supported by established youth motor development and sport specialization literature, which indicates that approximately six years of structured practice marks a transition from the fundamental skill acquisition phase to the specialization stage of athlete development [7]. Maturity status was assessed using the maturity offset method [26], which estimates the time before or after Peak Height Velocity (PHV) using current anthropometric measurements. No longitudinal growth-rate data were used. Maturity offset values ranged from –0.52 to 2.84 (1.30 ± 0.88), reflecting a distribution from pre- to post-PHV. Participation in the study was voluntary, and all athletes, along with their legal guardians, provided informed consent before data collection. The study was conducted in accordance with the Declaration of Helsinki and approved by the Research Ethics Committee of the School of Physical Education and Sport Science of the Aristotle University of Thessaloniki (257/2025). A power analysis conducted using G*Power 3.1 confirmed that a sample size of 40 participants was sufficient to detect correlations between cognitive and motor variables, achieving a statistical power of 0.83 at a significance level of α < 0.05.

### 2.2. Experimental Design

Participants were evaluated using valid and reliable tests of specific motor and perceptual skills that were selected as critical for soccer performance. The evaluation was conducted during the athletes’ regular training time on the field, as the equipment was portable. The assessment took place over two days, as presented in Table 1. Cognitive assessments were conducted during the first testing day, while all motor tests (CMJ, sprint, and change-of-direction) were completed during the second testing session, scheduled 48 h later. Within each session, the order of tests was randomized across participants to minimize potential sequencing effects.

All measurements were conducted on the training fields where the athletes had scheduled practices. Before the tests began, the athletes performed a warm-up program including jogging and dynamic stretching exercises [27]. All athletes were familiar with the testing procedures before the beginning of the measurements. Initially, anthropometric characteristics were recorded: body mass, body height, sitting height, leg length, single arm reach test (MROA), both arms reach test (MRBA), and arm span. Below is a detailed description of the motor and perceptual tests:

Speed was assessed through a 10 m maximal sprint, and the time was recorded using three pairs of photocells placed at the starting point, at 5 m, and 10 m (Microgate, Bolzano, Italy). The participants started from a standing position, with their dominant foot 0.3 m behind the first photocell gate and sprinted maximally until passing the third gate. The photocells were positioned approximately 0.6 m above the ground and spaced 1.5 m apart.

To assess lower limb power, three vertical jumps were performed: the Squat Jump (SJ), the Countermovement Jump (CMJ), and the Drop Jump (DJ). In the SJ, the participant started from a semi-squat position (90° knee angle) and executed a maximal vertical jump without further knee flexion. In the CMJ, the participant, starting from a standing position, performed a quick preparatory movement by flexing the hips and knees to approximately 90°, followed by an explosive jump with full hip and knee extension. In DJ, participants stood on top of a box at a height of 20 cm with their feet shoulder-width apart. They were instructed to lean forward and drop from the box. Jumping instead of stepping off the box was not permitted. Upon ground contact, players were instructed to minimize ground contact time and immediately perform a maximum vertical jump and then land on the floor and stick the landing in line with previous recommendations (1). During execution, the arms remained on the hips. All jumps were performed using an optical measurement system, OptoJump (Microgate, Bolzano, Italy), which has reported near-perfect reliability and validity [28].

Agility was assessed using the *t*-test. The participant started at will, behind point A, and sprinted 9.14 m to point B, touching a 30 cm cone with the right hand. Then, they shuffled 4.57 m to the left to point C and touched the cone with the left hand. Next, they shuffled 9.14 m to point D and touched the cone with their right hand. They then shuffled 4.57 m left again to point B and touched the cone with the left hand. Finally, they backpedaled quickly to point A. Time was recorded using a photocell gate placed at point A (Microgate, Bolzano, Italy).

The semicircular test was used as an additional agility test in response to stimulus, including both a pre-planned (change of direction speed, SC-CODS) and a reactive agility (SC-RA) protocol. Both conditions were measured by the FitLight Trainer, a wireless reaction training system manufactured by Sport Corp. (Ontario, Canada), as described by Čoh and colleagues [29]. In the pre-planned protocol, all subjects knew the order of switching the LED light in the designated order: 1-2-3-4-5-6. In the RA condition, the order of LED activation was unknown and random. Participants were divided into two groups and alternated between protocols. They performed two trials, and the best result of each condition was retained as the final score. In addition, the REAC-INDEX, which represents the time differences between the SC-RA test result and the measurement of SC-CODS of a similar pattern and for similar distances [30], was determined. REAC – INDEX[s] = RA[s] − CODS [s].

The perceptual skills assessed were psychomotor speed, visuospatial working memory, and spatial visualization, using evaluation criteria based on the correctness of the answer (%) and the reaction time (msec) to the correct answers. In the present study, the selection of perceptual–cognitive measures—visuospatial working memory, spatial visualization, and psychomotor speed—was guided by their established relevance to key soccer-specific demands [31,32]. These functions underpin core processes such as anticipation, tactical decision-making, pattern recognition, and visual search efficiency, all of which are essential in rapidly changing game environments [33]. Previous research in invasion sports has shown that athletes with stronger executive and perceptual–cognitive skills outperform their peers in tasks requiring rapid information processing and situational awareness, supporting the inclusion of these specific tests in the current protocol [31]. These skills were evaluated using a laptop equipped with E-PRIME 3.0 software and the specially designed keyboard, Chronos. Each test lasted 1–2 min and was user-friendly. Ιn more detail:

Assessment of visuospatial memory is conducted through the “Corsi Block-Tapping Task”, which evaluates visual–spatial short-term working memory in participants. In this procedure, individuals observe a sequence of nine blocks displayed on a screen, with a series of squares highlighted in yellow. After the sequence is presented, participants must replicate the order of the squares and indicate completion by clicking “Done.” Female athletes begin with a sequence of two squares, and with each correct response, the length of the subsequent sequence increases by one. If both sequences are answered correctly, the length remains unchanged; however, if both are incorrect, the sequence length decreases by one. The assessment comprises fourteen trials, during which no feedback is provided. This methodology facilitates the measurement and evaluation of memory capabilities, as athletes are tasked with observing and reproducing sequences of illuminated squares, with the complexity escalating according to their performance. The test captures both the speed and accuracy of responses, yielding comprehensive data on participant performance.

The assessment of spatial visualization involves a second test focused on sensation and perception. In this E-prime’s test, “Rotations of Mental Images Test”, athletes are shown a letter on a screen and must determine whether it is displayed in its standard orientation, as a mirror image, or in an inverted form. In half of the instances, the letter appears in its usual format, while in the other half, it is mirrored. The cognitive mental image rotation test is essential for evaluating spatial intelligence, which refers to the capacity to comprehend and analyze intricate spatial relationships and to interpret mental spatial data. Proficient spatial visualization enables athletes to anticipate the trajectory of the ball and to grasp the arrangement of players on the field, thereby providing them with a cognitive edge. Again, the test captures both the speed and accuracy of responses, yielding comprehensive data on participant performance.

The evaluation of psychomotor speed is conducted through E-prime’s “Simple Reaction Time Task Test”, which is designed to gauge the fundamental cognitive and motor responses of participants by measuring their rapidity in reacting to visual stimuli. During this assessment, athletes are instructed to promptly press the “1” key upon viewing an image of a star displayed on the screen. The duration from the appearance of the stimulus to the recording of the reaction is measured, thereby providing an assessment of their psychomotor speed. Again, the test captures both the speed and accuracy of responses, yielding comprehensive data on participant performance.

### 2.3. Statistical Analysis

Descriptive statistics were initially used to highlight some additional parameters according to training age, age, maturity, perceptual, and motor skills. Means, standard deviations, minimum, maximum, and percentage were displayed. To identify the relationships between perceptual (Hypothesis 1) and motor skills (Hypothesis 2) in relation to training age, age, and maturity, the Pearson Correlation and Independent *t*-test were used. Specifically, the Pearson Correlation was used to identify possible correlations between maturity and cognitive and motor skills, while the Independent *t*-test was used to investigate differences between the groups of training age and age with cognitive and motor skills. Both sporting experience and participants’ age were considered in the analyses; however, preliminary results indicated that age did not have a statistically significant effect. Before performing the Pearson correlation analyses, assumptions of approximate normality and absence of influential outliers were evaluated using visual inspection of histograms, Q–Q plots, and standardized residual diagnostics. For the third hypothesis, which was to identify if cognitive skills can influence motor skills and if training age, age, and maturation can predict perceptual and motor skills, Multiple Linear Regression was used. Adjusted R-squared (_ADJ_R^2^) was also used to identify the total variance explained by each model. All statistical analyses were conducted using IBM SPSS 28.0 Statistics with level of significance of *p* < 0.05.

## 3. Results

The athletes’ heights varied between 1.47 and 1.89 (1.72 ± 0.09), their weights ranged from 38.10 to 100.40 (66.06 ± 12.31), and their sitting heights ranged from 79 to 99 (90.15 ± 5.05). The athletes’ leg length varied between 68 and 94 (82.61 ± 5.13), their MROA ranged between 1.88 and 2.42 (2.24 ± 0.11), their MRBA was between 1.85 and 2.40 (2.21 ± 0.11), and their arm span was between 1.45 and 1.91 (1.74 ± 0.09). Finally, their training age was between 1 and 12 years of training (7.43 ± 3.09), their age was between 12.52 and 16.71 (14.86 ± 0.81), and their maturity was between −0.52 and 2.84 (1.30 ± 0.88). Descriptive statistics for the athletes’ training age, age, and maturity offset are presented in Table 2.

Participants completed a series of perceptual assessments in psychomotor speed, visuospatial working memory, and spatial visualization skills, evaluating the accuracy of their responses (% of correct answers), and their reaction time (msec). Their performance was summarized using means, standard deviations, minimum, maximum, and percentages.

For the visuospatial working memory assessment, participants achieved a minimum value of 6 correct answers and a maximum value of 10 correct answers (8.59 ± 1.14), scoring 85.9%. Concerning their reaction time, a minimum value of 1415.80 ms and a maximum value of 3974.50 ms were displayed (2627.38 ± 620.92).

For the psychomotor speed assessment, the average reaction time was 255.96 ms (SD = 22.16), with a minimum value of 203.22 ms and a maximum value of 295.28.

For the spatial visualization assessment, participants scored a minimum value of 14 correct answers and a maximum value of 24 correct answers (18.49 ± 2.44), scoring 77%. Regarding reaction time in the same test, there was a minimum value of 508.12 ms and a maximum value of 1094.95 (789.82 ± 158.96). This information is represented in Table 3.

Participants also completed a series of motor tests evaluating their ability in the Sprint and Change of Direction tasks. Their performance is summarized in Table 4 using means, standard deviations, minimum, maximum, and 95% CI mean upper and lower.

In the sprinting tests, times for the 5 m sprint ranged from 0.95 to 1.35 s (1.15 ± 0.08), indicating highly consistent performance. The 10 m sprint ranged from 1.74 to 2.35 s, (1.97 ± 0.12), while their performance for SJ ranged from 15.30 to 36.10 cm (27.43 ± 5.16); for CMJ their performance varied from 18.30 to 39.50 cm (29.54 ± 5.43), and for the DJ their performance showed a minimum value of 14.60 cm and a maximum value of 36.30 cm (27.05 ± 5.04). Their agility in the *t*-test ranged from 9.40 to 11.85 s (10.28 ± 0.62). In the CODS protocol of the semicircular test, participants recorded times ranging from 9.22 to 13.22 s (11.24 ± 1.08), and for the reactive configuration (SC-RA), times ranged from 12.26 to 18.14 s (14.5 ± 1.25). For the REAC-INDEX, the values ranged from 1.45 to 6.83 (3.26 ± 1.16).

To identify correlations between maturity and perceptual tests and motor tests, the Pearson Correlation was utilized. The correlation analysis did not reveal statistically significant relationships. There was no statistically significant correlation between maturity and visuospatial working memory assessment (Correct answers), r = −0.12, *p* = 0.45, and Reaction Time, with r = 0.12, *p* = 0.45. Psychomotor speed assessment (Reaction Time) did not show any statistically significant correlation with age, r = 0.27, *p* = 0.09. There was also no statistically significant correlation between age and spatial visualization assessment (Correct Answers), r = 0.01, *p* = 0.97, and Reaction Time, r = 0.18, *p* = 0.27.

For maturity, a statistically significant correlation is observed between sprint 5 m (r = −0.55, *p* < 0.001), sprint 10 m (r = −0.62, *p* < 0.001), and *t*-test performance (r = −0.55, *p* < 0.001). Statistically significant positive correlation was observed between age and SJ (r = 0.47, *p* < 0.01), CMJ (r = 0.51, *p* < 0.01), and DJ (r = 0.47, *p* < 0.01). A negative statistically significant correlation was found between age and pre-planned change of direction (r = −0.35, *p* < 0.05), whereas there was no statistically significant correlation with reactive agility (r = −0.26, *p* = 0.09). There was also no statistically significant correlation found between age and REAC-INDEX (r = 0.04, *p* = 0.80). This information is displayed in Table 5.

**Figure 1:**
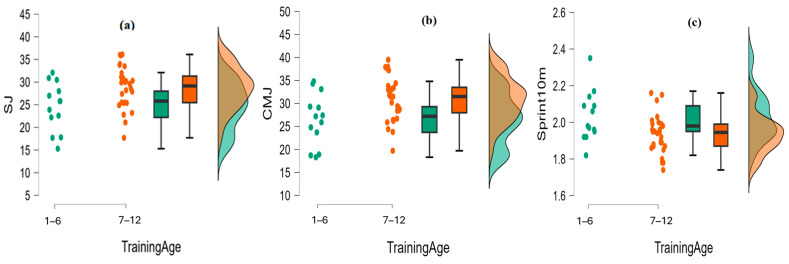
(**a**) Raincloud plot for training age and Squat Jump. (**b**) Raincloud plot for training age and Countermovement Jump. (**c**) Raincloud plot for training age and Sprint 10 m.

The independent *t*-test was employed to identify any differences between the two groups in training age concerning cognitive and motor skills. Statistically significant differences were only found between training age and SJ, CMJ, and Sprint 10 m. Specifically, statistically significant differences were found between training age and SJ, t(38) = −2.476, *p* < 0.05, Cohen’s *d* = −0.86. The 1–6 years of training age group (24.58 ± 5.33) presented worse performance than the 7–12 years of training age group (26.58 ± 5.67). Moreover, statistically significant differences were found between training age and CMJ, t(38) = −2.447, *p* < 0.05, Cohen’s *d* = −0.85. It was found again that the 1–6 years of training age group (26.58 ± 5.67) presented worse performance than the 7–12 years of training age group (30.83 ± 4.89). Statistically significant differences were also found between training age and Sprint in 10 m, t (38) = 2.484, *p* < 0.05, Cohen’s *d* = −0.62. The group with more years of experience (1.93 ± 0.11) presented better performance than the group with less experience (2.03 ± 0.14). This information is displayed in Figure 1.

**Figure 2:**
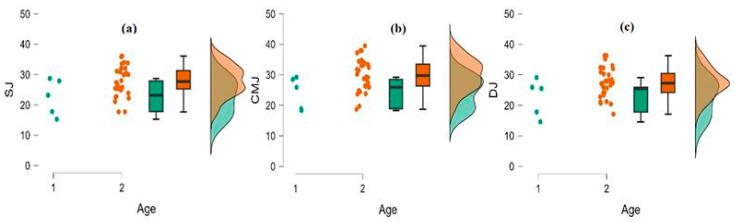
(**a**) Raincloud plot for age and Squat Jump. (**b**) Raincloud plot for age and Countermovement Jump. (**c**) Raincloud plot for age and Drop Jump (1: U14, 2: U16).

The independent *t*-test was also employed to identify any differences between the two groups in terms of age, concerning cognitive and motor skills. Statistically significant differences were only found between training age and SJ, CMJ, and DJ. Specifically, statistically significant differences were found between age and SJ, t(35) = −2.184, *p* < 0.05, Cohen’s *d* = −1.05. The U14 (22.58 ± 5.96) presented worse performance than the U16 (27.97 ± 5.02) in SJ. Statistically significant differences were also found between age and CMJ, t(35) = −2.316, *p* < 0.05, Cohen’s *d* = −1.11. Specifically, it was found that the U14 (24.16 ± 5.23) again presented worse performance than the U16 (29.96 ± 5.21) in CMJ. Furthermore, statistically significant differences were found between age and DJ, t(35) = −2.038, *p* < 0.05, Cohen’s *d* = −0.98. The U14 (22.58 ± 6.09) again presented worse performance than the U16 (27.39 ± 4.73) in DJ. This information is displayed in Figure 2.

**Figure 3:**
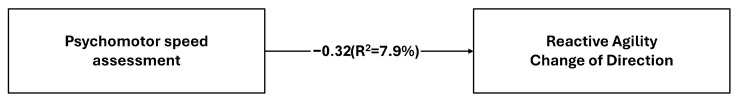
Psychomotor speed assessment influence on Reactive Agility Change of Direction.

Linear regression was used in order to investigate whether the cognitive tests influence motor skills. The analyses showed that only the reaction time of psychomotor speed assessment presented a statistically significant influence on the reactive agility of change of direction speed, F(1,40) = 4.419, *p* < 0.05. Specifically, there was a negative influence β = −0.32 (t = −2.102, *p* < 0.05). The total distribution that is explained from the specific model is _Adj_R^2^ = 7.9%. This information is presented in Figure 3.

**Figure 4:**
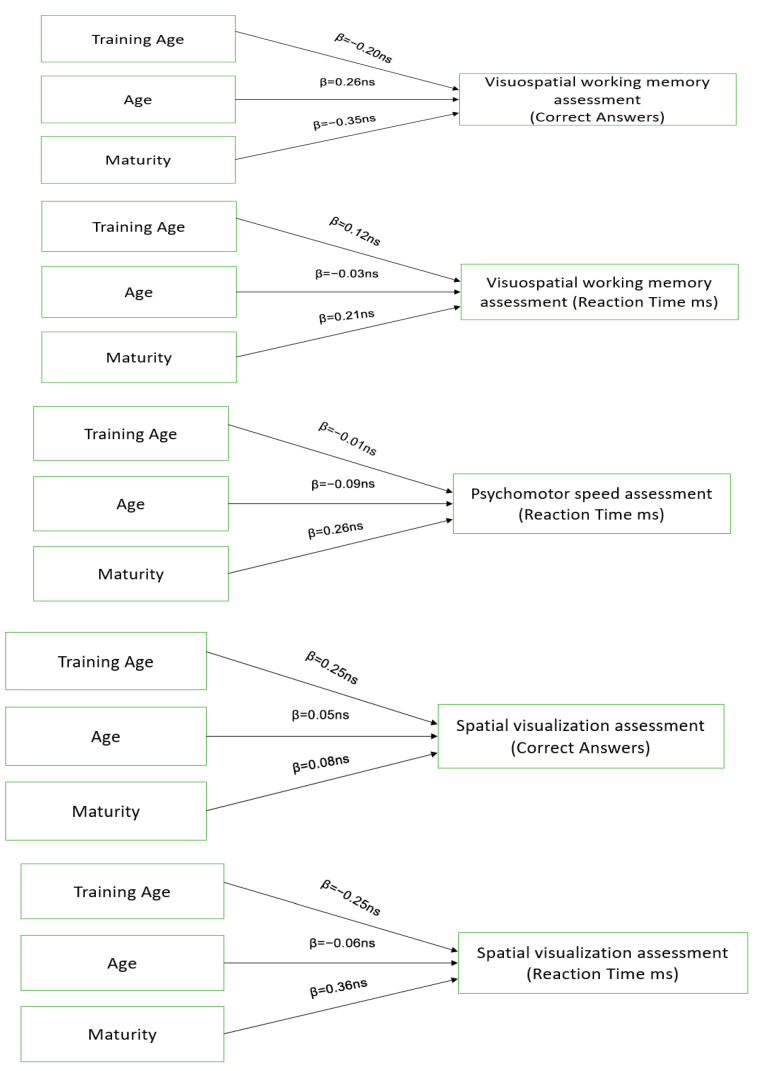
The effects of training age, age, and maturity on cognitive skills.

Training age, age, and maturity did not influence the Visuospatial working memory assessment (Correct Answers), F(3,35) = 1.522, *p* = 0.228, and Visuospatial working memory assessment (Reaction Time ms), F(3,35) = 0.600, *p* = 0.620. The three independent variables did not influence Psychomotor speed assessment (Reaction Time ms), F(3,35) = 0.551, *p* = 0.651. Training age, age, and maturity did not influence Spatial visualization assessment (Correct Answers), F(3,35) = 0.906, *p* = 0.449, and Spatial visualization assessment (Reaction Time ms), F(3,35) =2.117, *p* = 0.118. This information is presented in Figure 4.

Linear Regression analysis was utilized to identify if age, training age, and maturity may predict perceptual and motor skills. It was found that age, training age, and maturity influenced Sprint performance in 5 m, with F(3,35) = 4.645, *p* < 0.01. Specifically, maturity was a negative predictor, indicating that higher maturity levels were associated with faster sprint times. The standardized regression coefficient was β = −0.53 (t = −2.89, *p* < 0.011). Training age β = −0.20 (t = −1.34, *p* = 0.181) and age β = 0.03 (t = 0.18, *p* = 0.855) do not have a statistically significant influence. The model explained 23.8% of the variance (_Adj_R^2^ = 0.238). It was also found that age, training age, and maturity affected Sprint performance in 10 m, with F(3,35) = 8.104, *p* < 0.001. Specifically, training age β = −0.32 (t = −2.38, *p* < 0.05) and maturity =−0.61 (t = −3.71, *p* < 0.01) were negative predictors, indicating that higher training age and maturity were associated with faster sprint times. Age β = 0.08 (t = 0.47, *p* = 0.642) does not have a statistically significant influence. The model explained 37.8% of the variance (_Adj_R^2^ = 0.378). It was also presented that age, training age, and maturity influenced the *t*-test, with F(3,35) = 4.469, *p* < 0.01. Specifically, maturity = −0.55 (t = −2.98, *p* < 0.01) was a negative predictor, indicating that higher maturity was associated with better agility. Training age β = −0.15 (t = −1.01, *p* = 0.322) and age β = 0.05 (t = 0.26, *p* = 0.798) do not have a statistically significant influence. The model explained 22.9% of the variance (_Adj_R^2^ = 0.229). Age, training age, and maturity also influenced SJ, with F(3,35) = 6.466, *p* < 0.01. Specifically, training age β = 0.34 (t = 2.46, *p* < 0.05) and maturity β = 0.46 (t = 2.69, *p* < 0.05) were positive predictors, indicating that higher training age and maturity were associated with better performance in SJ. Age β = 0.06 (t = 0.35, *p* = 0.728) does not have a statistically significant influence. The model explained 31.9% of the variance (_Adj_R^2^ = 0.319). Age, training age, and maturity also influenced CMJ, with F(3,35) = 6.247, *p* < 0.01. Specifically, training age β = 0.33 (t = 2.33, *p* < 0.05) and maturity β = 0.46 (t = 2.57, *p* < 0.05) were positive predictors, indicating that higher training age and maturity were associated with better performance in CMJ. Age β = 0.09 (t = 0.52, *p* = 0.610) does not have a statistically significant influence. The model explained 31% of the variance (_Adj_R^2^ = 0.310). Age, training age, and maturity also influenced DJ, with F(3,35) = 4.255, *p* < 0.05. Specifically, maturity β = 0.45 (t = 2.42, *p* < 0.05) was a positive predictor, indicating that higher maturity was associated with better performance in DJ. Training age β = 0.23 (t = 1.51, *p* = 0.142) and age β = 0.05 (t = 0.28, *p* = 0.780) do not have a statistically significant influence. The model explained 21.8% of the variance (_Adj_R^2^ = 0.218). Age, training age, and maturity did not influence the pre-planned change of direction, reactive agility, and REAC-INDEX, with F(3,35) = 1.539, *p* = 0.223, F(3,35) = 0.684, *p* = 0.568, and F(3,35) = 0.614, *p* = 0.611, respectively (Table 6).

## 4. Discussion

The present study investigated how biological maturation, chronological age, and training experience interact to influence both motor and perceptual–cognitive performance in adolescent soccer players. The findings demonstrate that biological maturation and years of training experience are strong determinants of physical performance, whereas perceptual–cognitive skills appear to follow a more independent developmental trajectory. Among all cognitive variables assessed, psychomotor speed was the only factor associated with reactive agility, emphasizing the unique role of rapid information processing in dynamic sport-specific contexts. These results align with previous evidence showing that youth sport strongly supports improvements in physical capacities, but that cognitive skills require targeted and systematic training to develop meaningfully [19].

One of the principal findings is that biological maturation significantly predicted sprint performance over 5 m and 10 m, whereas chronological age did not. This pattern supports earlier reports indicating that biological maturity explains variability in sprint capacity more effectively than chronological age in young athletes [34,35]. Increases in muscle mass, neuromuscular efficiency, stride length, and coordination that occur during puberty likely contribute to the superior sprint performance of more mature players [36,37,38]. Considering that straight-line sprinting is among the most decisive actions during critical match situations [39], these findings highlight the importance of accounting for biological maturity in talent identification processes. Without such consideration, temporary advantages associated with advanced maturation may be mistaken for long-term athletic potential.

Moreover, these results underscore the practical implications of maturity-related advantages in talent identification. Early-maturing athletes often demonstrate superior sprinting and power-based capacities due to transient biological advantages, which may lead coaches to interpret these differences as indicators of superior talent. This creates a well-documented risk of systematic over-selection of early maturers, a bias that has been widely observed in youth soccer and other team sports [40,41]. Consequently, late-maturing athletes—who may possess strong perceptual–cognitive or technical abilities—are at risk of being overlooked, resulting in reduced developmental opportunities and increased dropout rates [1]. Integrating biological maturity monitoring into talent identification systems is therefore essential to ensure that short-term physical advantages are not mistaken for long-term athletic potential. Biological maturity was also associated with performance in pre-planned agility, vertical jump tests, and the *t*-test. These results align with earlier research describing the influence of maturation on neuromuscular power, coordination, and explosive movement patterns [7,42]. Such physical attributes support rapid accelerations, decelerations, and changes of direction—actions that are fundamental to soccer performance.

The maturity-related effects observed in sprinting, jumping, and pre-planned agility also align closely with the recent literature examining the relationship between maturation and performance in youth athletes. Contemporary studies consistently report that biological maturity is strongly associated with neuromuscular power and overall physical performance in adolescent team-sport players [43,44,45]. These findings reinforce the view that maturation drives substantial inter-individual differences in physical capabilities during adolescence, whereas perceptual–cognitive skills appear to follow more independent developmental pathways shaped predominantly by training specificity rather than biological growth [31]. The current results, therefore, strengthen recent evidence supporting the need to disentangle maturation-driven performance variation from trainable athletic qualities when evaluating youth players.

In contrast, biological maturity did not relate to reactive agility, suggesting that reactive agility depends primarily on perceptual and decision-making processes rather than on physical maturation [46]. This dissociation reinforces the broader understanding that participation in sport promotes not only physical development but also perceptual and cognitive engagement, both of which are important for overall health and development [47]. Reactive agility requires athletes to process unpredictable stimuli and rapidly select an appropriate motor response, distinguishing it from pre-planned agility tasks [48]. The absence of a maturation effect indicates that reactive agility does not improve simply as a result of physical growth. Instead, these skills appear to depend upon perceptual–motor coupling, attention, and decision-making. This finding underscores the need for training programs that incorporate cognitively demanding drills when the goal is to enhance reactive agility, as improvements in physical capacities alone are insufficient [49].

Training age emerged as another important determinant of motor performance. Players with longer training experience performed better in sprinting and jumping tasks compared with players who had fewer years of structured soccer practice. This observation corresponds to evidence that prolonged exposure to systematic training enhances neuromuscular coordination, strength, and motor skill execution [41,50]. Given the importance of acceleration, explosive jumping, and rapid directional changes in soccer [40], these results highlight the value of long-term training programs for athletic development. Furthermore, the developmental period of adolescence offers a window during which structured sport participation contributes to physical literacy and promotes long-term engagement in healthy physical activity habits [41].

Among perceptual–cognitive variables, psychomotor speed was the only predictor of reactive agility. Neither visuospatial working memory nor spatial visualization demonstrated significant associations with motor performance. Additionally, perceptual–cognitive outcomes were not influenced by maturation, age, or training experience. A plausible explanation for the absence of associations between training age or biological maturity and the cognitive variables is that physical and cognitive development follow partly independent trajectories during adolescence. Physical performance benefits directly from the hormonal, neuromuscular, and structural adaptations that occur during puberty, whereas improvements in executive and perceptual–cognitive skills depend largely on the quality and specificity of training experiences rather than on maturation itself [31]. Game-relevant cognitive skills such as anticipation, pattern recognition, and rapid decision-making are typically enhanced through targeted and cognitively rich practice conditions, rather than through general sport participation alone [19]. Consequently, unless young players are exposed to structured perceptual–cognitive training, differences in executive functioning may not become sufficiently pronounced to emerge in group comparisons. This interpretation aligns with contemporary evidence supporting holistic athlete development models in which maturity status, prior training exposure, and perceptual–cognitive capabilities are jointly considered to reduce selection bias, promote long-term retention, and support athletes’ overall health and well-being [32]. The relationship between psychomotor speed and reactive agility supports previous research emphasizing the importance of rapid processing speed and decision-making in time-constrained sports environments [10,11,51]. Quick reactions appear to facilitate efficient integration of perceptual cues with appropriate motor actions, explaining their predictive role in reactive agility performance.

The absence of associations between executive functions and motor outcomes contrasts with studies showing that working memory, cognitive flexibility, and inhibitory control differentiate elite from sub-elite youth athletes [13,52]. A plausible explanation is that the players in the present study may have had limited exposure to structured cognitive training. Recent research suggests that without explicit perceptual–cognitive training, such as VR-based decision tasks or video-based anticipation practice, youth athletes may not show measurable improvements in executive functioning [16,53,54]. While physical and technical training are regularly integrated into soccer practice, the development of perceptual–cognitive skills often occurs incidentally and without explicit instructional design. As a result, differences in executive functioning may not have been sufficiently pronounced to emerge in this sample [19,55].

The overall pattern of findings corresponds to the Youth Physical Development Model [7], which posits that physical and cognitive abilities follow different developmental pathways. Biological maturation is closely tied to improvements in strength, speed, and power, whereas perceptual–cognitive skills appear to be more strongly shaped by training quality and experience rather than training duration alone [47,56]. Previous work suggests that perceptual–cognitive expertise develops through high-quality, context-specific practice rather than through generic exposure to sport [57]. The lack of associations between training age and cognitive outcomes in this study supports the possibility that the nature of training—specifically, whether it includes cognitively demanding elements—is more influential than simply the number of years an athlete has participated [20].

Taken together, the findings demonstrate the distinct but complementary roles of biological maturation and training experience in shaping youth athletic development. While physical performance is strongly influenced by maturation-related changes and accumulated training exposure, psychomotor components of cognition also contribute uniquely to performance in unpredictable contexts. This holistic perspective underscores the importance of evaluating both motor and cognitive domains when assessing youth athletes. Encouraging balanced development during adolescence may have benefits beyond sport performance, supporting psychological resilience, cognitive engagement, and long-term participation in physical activity [25].

Several limitations should be noted. The cross-sectional design restricts the ability to infer causal relationships, and longitudinal studies would be valuable for understanding how physical and cognitive abilities evolve across maturation. Furthermore, the relatively small sample size, which consisted exclusively of male youth soccer players at a similar competitive level, may limit the generalizability of the results. Furthermore, training age was based on self-reported years of participation rather than on detailed information about training content or cognitive load. Future studies should include training quality metrics to clarify how specific types of practice shape both motor and cognitive development.

From a practical perspective, the results emphasize the importance of integrating maturity assessments and perceptual–cognitive evaluations into youth soccer development programs. Coaches may unintentionally overlook late-maturing athletes who possess strong perceptual–cognitive skills [23]. Training strategies should be tailored not only to physical characteristics but also to the cognitive profiles of players. Incorporating drills that enhance reaction time, anticipation, pattern recognition, and decision-making under pressure may yield substantial benefits. Emerging technologies such as virtual reality and interactive perceptual training tools offer promising avenues for implementing ecologically valid, cognitively demanding training environments [19,24]. These approaches can help ensure that players receive balanced support across physical and cognitive domains, thereby promoting both immediate performance and long-term development.

## 5. Conclusions

This study indicates that biological maturation and training experience contribute meaningfully to sprinting, jumping, and pre-planned agility performance in youth soccer players, whereas their influence on perceptual–cognitive outcomes appears limited. Psychomotor speed emerged as the only cognitive factor associated with reactive agility, highlighting the importance of rapid information processing in unpredictable match scenarios.

Overall, the findings suggest that motor and perceptual–cognitive abilities may develop along partly independent pathways during adolescence. While maturation supports improvements in physical performance, enhancements in perceptual–cognitive skills may require more targeted and structured training stimuli. These observations underscore the value of development frameworks that consider maturity status, training history, and cognitive profiles when evaluating or supporting young athletes. Such an approach may also help minimize selection biases that favor early maturers and promote more sustainable athlete development.

From a health and well-being perspective, fostering both motor and cognitive development within youth sport may contribute positively to sustained physical activity participation, psychosocial growth, and the reduction in inactivity-related health risks [25,58]. Future research should continue to examine how the content and quality of training—particularly cognitively demanding tasks—shape the development of executive functions in young players. The use of emerging technologies may offer promising tools for systematically training perceptual–cognitive skills and strengthening the integration between physical and cognitive development.

## Figures and Tables

**Table 1:** Experimental procedures.

Schedule	Experimental Procedures
Day 1	-Anthropometry (body mass, body height, sitting height, leg length, single arm reach test (MROA), both arms reach test (MRBA), and arm span)
	-Assessment of perceptual skills (psychomotor speed, visuospatial working memory, and spatial visualization)
	-Assessment of reactive agility (RA) and change of direction speed (CODS) via the semicircular test (SC)
Day 2	-Assessment of speed (5 m and 10 m)
	-Lower limb power assessment (SJ-Squat Jump, CMJ-Countermovement Jump, and DJ20-Drop Jump from a height of 20 cm)
	-Agility assessment (*t*-test)

**Table 2:** Mean, standard deviations, minimum, maximum, and 95% CI mean upper–lower for anthropometric characteristics, training age, age, and maturity.

	Minimum	Maximum	Mean ± SD	95% CI Mean Upper	95% CI Mean Lower
Height	1.47	1.89	1.72 ± 9.10	1.75	1.69
Body mass	38.10	100.40	66.06 ± 12.31	69.94	62.17
Sitting height	79	99	90.15 ± 5.05	91.74	88.55
Leg length	68	94	82.61 ± 5.13	84.23	80.99
MROA	1.88	2.42	2.24 ± 0.11	2.28	2.21
MRBA	1.85	2.40	2.21 ± 0.11	2.25	2.18
Arm Span	1.45	1.91	1.74 ± 0.09	1.77	1.71
Training age	1	12	7.43 ± 3.09	8.40	6.45
Age	12.52	16.71	14.86 ± 0.81	15.12	14.60
Maturity	−0.52	2.84	1.30 ± 0.88	1.58	1.03

**Table 3:** Mean, standard deviations, minimum, maximum, percentage, and 95% CI mean upper–lower for cognitive tests.

Assessments	Minimum	Maximum	Mean ± SD	% *	95% CI Mean Upper	95% CI Mean Lower
Visuospatial working memory assessment (Correct Answers)	6	10	8.59 ± 1.14	85.9%	8.95	8.23
Visuospatial working memory assessment (Reaction Time ms)	1415.80	3974.50	2627.38 ± 620.92		2823.37	2431.39
Psychomotor speed assessment (Reaction Time ms)	203.22	295.28	255.96 ± 22.16		262.95	248.96
Spatial visualization assessment (Correct Answers)	14	24	18.49 ± 2.44	77%	19.26	17.72
Spatial visualization assessment (Reaction Time ms)	508.12	1094.95	789.82 ± 158.96		840	739.65

Note. % *: The percentages correspond to the total percentage of the correct answers.

**Table 4:** Mean, standard deviations, minimum, maximum, and 95% CI mean upper–lower for motor rests.

	Minimum	Maximum	Mean ± SD	95% CI Mean Upper	95% CI Mean Lower
Sprint 5 m (s)	0.95	1.35	1.15 ± 0.08	1.17	1.12
Sprint 10 m (s)	1.74	2.35	1.97 ± 0.12	2.00	1.93
SJ (cm)	15.30	36.10	27.43 ± 5.16	29.06	25.80
CMJ (cm)	18.30	39.50	29.54 ± 5.43	31.25	27.82
DJ20 (cm)	14.60	36.30	27.05 ± 5.04	28.65	25.46
*t*-test (sec)	9.40	11.85	10.28 ± 0.62	10.47	10.08
SC-CODS(s)	9.22	13.22	11.24 ± 1.08	11.58	10.90
SC-RA (s)	12.26	18.14	14.5 ± 1.25	14.90	14.11
REAC-INDEX (s)	1.45	6.83	3.26 ± 1.16	3.62	2.89

SJ, Squat Jump; CMJ, Countermovement Jump; DJ20, Drop Jump from a 20 cm-high box; *t*-test, agility test; SC-CODS, time in the pre-planned condition of the semicircular test; SC-RA, time in the random condition of the semicircular test; REAC-INDEX, difference SC-CODS vs. SC-RA.

**Table 5:** Correlations between maturity and cognitive and motor skills.

	Visuospatial Working Memory Assessment (Correct Answers)	Visuospatial Working Memory Assessment (Reaction Time ms)	Psychomotor Speed Assessment (Reaction Time ms)	Spatial Visualization Assessment (Correct Answers)	Spatial Visualization Assessment (Reaction Time ms)				
**Maturity**	−0.12 ns	0.12 ns	0.27 ns	0.01 ns	0.18 ns				
	Sprint 5 m	Sprint 10 m	*t*-test	SJ	CMJ	DJ20	SC-CODS	SC-RA	REAC-INDEX
	−0.55 ***	−0.62 ***	−0.55 ***	0.47 **	0.51 **	0.47 **	−0.35 *	−0.26	0.04 ns

Note: * Correlation is significant at the 0.01 level. ** Correlation is significant at the 0.01 level. *** Correlation is significant at the 0.001 level. ns; non-significant. S J, Squat Jump; CMJ, Countermovement Jump; DJ20, Drop Jump from a 20 cm-high box; *t*-test, agility test; SC-CODS, time in the pre-planned condition of the semicircular test; SC-RA, time in the random condition of the semicircular test; REAC-INDEX, difference SC-CODS vs. SC-RA.

**Table 6:** Regression analysis parameters among training age, age, and maturity on motor skills.

Independent Variables	Dependent Variables	F	*p*	β	R^2^
Training age	Sprint 5 m	4.645	0.01	−0.20	23.8%
Age				0.03 ns	
maturity				−0.53 **	
Training age	Sprint 10 m	8.104	0.000	−0.32 *	37.8%
Age				0.08 ns	
maturity				−0.61 **	
Training age	*t*-test	4.469	0.01	−0.15	22.9%
Age				0.05 ns	
maturity				−0.55 **	
Training age	SJ	6.466	0.01	0.34 *	31.9%
Age				0.06 ns	
maturity				0.46 *	
Training age	CMJ	6.247	0.01	0.33 *	31%
Age				0.09 ns	
maturity				0.46 **	
Training age	DJ20	4.255	0.02	0.23 ns	21.8%
Age				0.05 ns	
maturity				0.45 *	
Training age	SC-CODS	1.539	0.223	−0.20 ns	4.4%
Age				−0.23 ns	
maturity				−0.08 ns	
Training age	SC-RA	0.684	0.568	−0.14 ns	2.8%
Age				0.08 ns	
maturity				−0.24 ns	
Training age	REAC-INDEX	0.614	0.611	0.03 ns	3.4%
Age				0.28 ns	
maturity				−0.18 ns	

Note: * Correlation is significant at the 0.05 level. ** Correlation is significant at the 0.01 level. ns; non-significant. SJ, Squat Jump; CMJ, Countermovement Jump; DJ20, Drop Jump from a 20 cm-high box; *t*-test, agility test; SC-CODS, time in the pre-planned condition of the semicircular test; SC-RA, time in the random condition of the semicircular test; REAC-INDEX, difference SC-CODS vs. SC-RA.

## Data Availability

The data that support the results of this study are available from the corresponding author upon reasonable request, as they cannot be shared publicly due to ethical restrictions.

## Abbreviations

The following abbreviations are used in this manuscript:

PHV	Peak Height Velocity
SJ	Squat Jump
CMJ	Countermovement Jump
DJ	Drop Jump
SC-CODS	Time in the pre-planned condition of the semicircular test
SC-RA	Time in random condition of the semicircular test
REAC-INDEX	Difference SC-CODS vs. SC-RA.
